# A Naturally Occurring Novel Reassortant Strain of Infectious Bursal Disease Virus from Quails

**DOI:** 10.3390/vetsci13090923

**Published:** 2026-09-08

**Authors:** Pengyu Guo, Xinyao Guo, Chun Yu, Wei Gao, Quan Xie, Tuofan Li, Zhimin Wan, Aijian Qin, Hongxia Shao, Jianqiang Ye

**Affiliations:** 1 Key Laboratory of Jiangsu Preventive Veterinary Medicine, Key Laboratory for Avian Preventive Medicine, Ministry of Education, College of Veterinary Medicine, Yangzhou University, Yangzhou 225009, China; 2 Jiangsu Co-Innovation Center for Prevention and Control of Important Animal Infectious Diseases and Zoonoses, Yangzhou 225009, China; 3 Joint International Research Laboratory of Agriculture and Agri-Product Safety, Ministry of Education, Yangzhou University, Yangzhou 225009, China; 4 Jiangsu Key Laboratory of Zoonosis, Yangzhou University, Yangzhou 225009, China; 5 Qingdao Phagepharm Bio-Tech Co., Ltd., Qingdao 266000, China

**Keywords:** IBDV, novel naturally reassortant, isolation, replication, quails

## Abstract

Infectious bursal disease virus (IBDV) primarily triggers severe immunosuppression in birds and has resulted in substantial economic losses to the poultry industry globally. Here, a novel naturally reassortant IBDV strain, designated IBDV_G3, was isolated from quails during a disease outbreak in Jiangsu Province, China. Genome sequencing revealed that IBDV_G3 carried segment A derived from the serotype-2-related A0 lineage, while segment B clustered within the B1a classical-like lineage, and this genomic constellation was further confirmed in the original uncultured quail tissues. IBDV_G3 replicated efficiently in LMH cells without prior adaptation, and its VP2 was recognized by the serotype 1 VP2-specific monoclonal antibody 5G10. These findings suggest that quails may harbor reassortant IBDV strains and that the quail–chicken interface warrants continuous surveillance for IBDV reassortment and evolutionary variation.

## Simple Summary

Infectious bursal disease (IBD) is a highly contagious viral infection that causes severe immunosuppression and major economic losses in poultry worldwide. The causative agent, IBDV, infects a wide range of birds, including wild species that can spread the virus without showing symptoms. In this study, we isolated a new reassortant IBDV strain, IBDV_G3, from diseased quails during an outbreak in Jiangsu Province, China. Genetic analysis showed that this strain carries a unique combination of genome segments: its segment A is related to serotype 2 IBDV, while its segment B belongs to the B1a classical-like lineage. IBDV_G3 replicated efficiently in cell culture without prior adaptation, and the same genomic combination was verified directly in the original quail tissues. Our findings suggest that quails may harbor such reassortant IBDV strains and that the quail–chicken interface deserves continuous surveillance for IBDV reassortment and evolutionary variation. Whether IBDV_G3 could serve as a vaccine candidate requires comprehensive future studies.

## 1. Introduction

Infectious bursal disease (IBD) is an acute, highly contagious viral disease of poultry caused by infectious bursal disease virus (IBDV). The infection primarily triggers severe immunosuppression in affected birds and has resulted in substantial economic losses for the global poultry industry [1,2]. IBDV is a member of the genus Avibirnavirus within the family Birnaviridae [3]. To date, IBDV has been divided into two distinct serotypes, serotype 1 and serotype 2. Specifically, serotype 1 is pathogenic to chickens, while serotype 2 is generally non-pathogenic for poultry species [4,5]. In accordance with the dual-segment genotyping system, IBDV strains are further classified into ten genotypes based on segment A (A1: classical strain; A2: US antigenic variant; A3: very virulent strain; A4: del-E IBDV (dIBDV); A5: atypical Mexican strain; A6: atypical Italian strain; A7: early Australian strain; A8: Australian variant strain; A9: Portuguese strain; A0: serotype 2 strain) and five genotypes based on segment B (B1: classical-like strain, including serotype 2, B1a, and B1b subtypes; B2: very virulent-like strain; B3: early Australian-like strain; B4: Polish and Tanzanian strains; B5: Nigerian strain) [6]. Notably, IBDV possesses a broad host spectrum, encompassing chickens, turkeys, ducks, guinea fowl, ostriches, and multiple wild bird species [7]. These wild birds can serve as asymptomatic carriers, facilitating the epidemiological circulation of IBDV [7,8]. In this study, a novel reassortant IBDV, designated as IBDV_G3, was isolated and characterized from diseased quails.

## 2. Materials and Methods

### 2.1. Virus Isolation

#### 2.1.1. Sample Collection and Processing

A total of 12 diseased quails showing depression, inappetence, and diarrhea were collected from five separate cages within the same quail farm in Jiangsu Province, which raised approximately 600,000 Coturnix japonica of about 60 days of age. During the outbreak, the affected flock exhibited a morbidity rate of 5% and a mortality rate of 1.5–2.5%. Infectious bursal disease (IBD) vaccination had not been included in the immunization program of this farm. Spleen and liver tissues were collected from each animal, and all tissue samples were processed individually without sample pooling. Collected tissues were homogenized in sterile phosphate-buffered saline (PBS) at a ratio of 1:10 (*w*/*v*). The homogenates were centrifuged at 5000 rpm for 15 min at 4 °C to remove cell debris, and the supernatants were filtered through a 0.22 μm filter to obtain cell-free suspensions for virus isolation. All operations of tissue collection, homogenate preparation, and sample processing were performed in a biological safety cabinet.

#### 2.1.2. Virus Isolation in LMH Cells

The prepared cell-free suspensions were inoculated onto LMH cell monolayers. Cells were incubated at 37 °C for 1 h to allow virus adsorption, after which the inoculum was discarded. Cells were washed once with PBS and overlaid with DMEM/F12 medium supplemented with 1% FBS. Cultures were maintained at 37 °C and monitored daily for cytopathic effects (CPE). When over 70% of the cell monolayer developed CPE including cell rounding, detachment and syncytium formation, cell culture supernatants were harvested. Harvested supernatants were subjected to three freeze-thaw cycles, clarified by centrifugation at 5000 rpm for 10 min, and stored at −80 °C.

#### 2.1.3. Plaque Purification and Genomic Validation

Virus plaque purification was performed using an agar overlay method. Virus stock were inoculated onto LMH cell monolayers. After virus adsorption, cells were overlaid with medium containing agar. Given that IBDV produces plaques of variable sizes, well-isolated single plaques were preferentially picked to avoid mixed-plaque contamination. Three successive rounds of plaque purification were conducted to generate clonal virus isolates. The final plaque-purified virus was subjected to Sanger sequencing of the VP2 gene to exclude cell-passage-introduced mutations. The passage-3 clonal virus was used for whole-genome sequencing and growth kinetics assay. To rule out mixed infection and laboratory contamination, Sanger sequencing was performed on both the original uncloned virus stock and independent plaque-purified clones, to verify the consistency of segment A and segment B.

#### 2.1.4. Routine Pathogen Screening

Liver homogenates were screened for common avian pathogens, including Fowl Adenovirus (FAdV), Infectious Bronchitis Virus (IBV), Avian Influenza Virus (AIV), Avian Reovirus (ARV), Mycoplasma Gallisepticum (MG), Mycoplasma Synoviae (MS), and Newcastle disease virus (NDV).

### 2.2. Morphological Identification

The supernatants of LMH cells inoculated with samples from quails were centrifuged at 12,000 r/min and 4 °C for 20 min to eliminate impurities. A total of 10 μL of the resulting supernatant was placed onto a copper grid and incubated at room temperature for 5 min. Then, 10 μL of a 2% phosphotungstic acid solution (pH 6.8) was applied to the copper grid for negative staining, which was allowed to proceed for 5 min. The prepared copper grid was examined using a transmission electron microscope (TEM, Model: JEM-1400Plus, JEOL, Akishima, Tokyo, Japan) operating at an acceleration voltage of 80 kV. The morphology, size, and distribution characteristics of the viral particles were observed, and representative fields of view were selected for photography and documentation.

### 2.3. Indirect Immunofluorescent Assay (IFA)

IFA was performed using the IBDV VP2-specific mouse monoclonal antibody 5G10 as the primary antibody, followed by FITC-conjugated goat anti-mouse IgG as the secondary antibody. The fluorescence signals were visualized under a fluorescence microscope.

### 2.4. Viral Growth Kinetics

Viral growth curves of the isolated IBDV strain were determined in LMH cells. The virus was inoculated at a multiplicity of infection (MOI) of 0.001 onto the cell monolayers, with three replicate wells prepared for each time point. Culture supernatants were collected at 24, 48, 72, 96 and 120 h post-infection (hpi). Viral titers in the collected supernatants were determined by the TCID_50_ assay on LMH cells. The TCID_50_ values were calculated using the Reed–Muench method. The viral growth curves were plotted with time post-infection (hpi) on the x-axis and viral titer (log_10_ TCID_50_/mL) on the y-axis using GraphPad Prism 9 software (version 9.5.0).

### 2.5. Whole Genome Amplification and Analysis

Total RNA extracted from infected cells was reverse-transcribed to cDNA, and the complete genome segments A and B were subsequently amplified using specific primers. The primer sequences used were as follows: Primers for segment A amplification: AF1 (5′-GGATACGATCGGTCTGACCCCGGGGGAGTC-3′), AR1 (5′-TGAAGTCGGTGTACTCCCTCG-3′), AF2 (5′-TTTAAGGACATCATCCGAGCC-3′), and AR2 (5′-GGGGACCCGCGAACGGATCCAATTTGGGAT-3′). Primers for segment B amplification: BF1 (5′-GGATACGATGGGTCTGACCCTCTGGGA-3′), BR1 (5′-TCTAGGTCAATTGAGTACCAC-3′), BF2 (5′-GTGGTACTCAATTGACCTAGA-3′), and BR2 (5′-GGGGGCCCCCGCAGGCGAAGGCCGGGGAT-3′). The PCR products of segments A and B were cloned into pMD19-T vectors (Takara Biotechnology Co., Ltd., Dalian, China) and the recombinant plasmids were sent to Tsingke Biotech (Nanjing) Co., Ltd. (Nanjing, China) for Sanger sequencing verification to ensure sequence accuracy. The sequencing results were used for subsequent whole-genome assembly.

The complete nucleotide sequences of segment A and segment B were obtained from the full-length genome. Multiple alignments of the complete segments along with refer-ence sequences retrieved from GenBank were prepared using Clustal X (version 2.0). Phylogenetic trees for complete segments A and B were constructed using the maximum likelihood (ML) method in IQ-TREE (version 3.1.2). The best-fit nucleotide substitution models were automatically determined by ModelFinder according to the Bayesian Information Criterion (GTR + F + I + G4 for segment A and TIM2 + F + I + G4 for segment B), and node support was evaluated with 1000 bootstrap replicates.

## 3. Results

**Figure 1:**
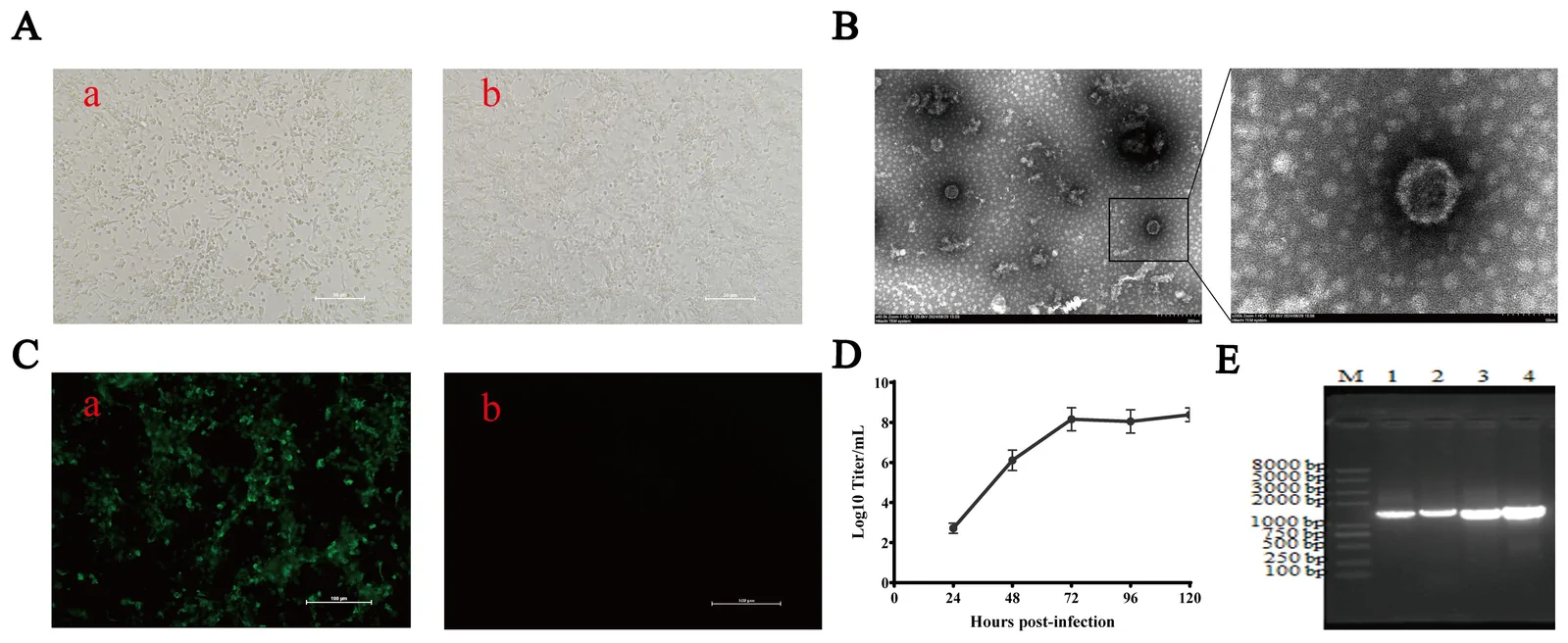
Identification and characteristics of a novel IBDV isolate (**A**). Cytopathic effect of LMH cells inoculated with the samples from diseased quails (**a**), and normal LMH cells without inoculation (**b**). (**B**) Morphological observation of the isolated viruses through TEM. (**C**) IFA detection of VP2 protein in LMH cells infected with IBDV_G3 (**a**), and normal LMH cells without infection (**b**). (**D**) Growth curve of IBDV_G3 in LMH cells, detailed replication kinetic data are shown in Table S1. (**E**) PCR amplification of the genome of IBDV_G3. Line M: 2000 bp marker; line 1: Segment A1 of IBDV_G3 (1400 bp); line 2: Segment A2 of IBDV_G3 (1800 bp); line 3: Segment B1 of IBDV_G3 (1364 bp); line 4: Segment B2 of IBDV_G3 (1500 bp) (The original PCR gel images can be found in Figure S3).

All 12 quail samples were tested and cultured independently in this study, with an individual IBDV-positive rate of 16.67% (2/12) among all sampled quails. To exclude common avian pathogens, the tissue samples were first screened by PCR for FAdV, IBV, AIV, ARV, MG, MS, and NDV, and all tested negative, demonstrating that the samples were free of mixed infection with these common avian pathogens. To isolate potential pathogenic agents, homogenate filtrates prepared from the spleen and liver tissues of affected quails were inoculated into LMH cell cultures. Typical cytopathic effects (CPE), characterized by cell shrinkage, rounding, partial cell detachment, and obvious syncytium formation, were observed at 48–72 h post-inoculation in LMH cells at the first inoculation passage (Figure 1A), whereas no CPE was detected in any negative control group or in the other sample inoculation groups, indicating viruses were successfully isolated from the positive liver tissue homogenates.

For further purification and identification, the viruses were purified via plaque assay, and viral morphology was subsequently examined by transmission electron microscopy (TEM). As shown in Figure 1B, the isolated viral particles exhibited a diameter of 60 nm, consistent with the typical morphological characteristics of IBDV. For molecular identification, a pair of specific primers targeting the IBDV VP5 gene was designed for PCR amplification, and a specific DNA fragment of approximately 450 bp was successfully amplified from LMH cells infected with the purified virus. Sanger sequencing confirmed that the amplified VP5 gene shared 97.3% nucleotide identity with the classical IBDV strain 23/82, verifying that the isolated pathogen was IBDV, which was designated IBDV_G3. Immunofluorescence assay (IFA) was further performed using the IBDV VP2-specific monoclonal antibody 5G10 for phenotypic identification. As presented in Figure 1C, distinct green immunofluorescence signals were detected in the cytoplasm of IBDV_G3-infected LMH cells, whereas no fluorescence was observed in the uninfected control cells. IBDV_G3 was capable of efficient replication in LMH cells without prior cell adaptation. For viral replication kinetics analysis, LMH cells were infected with IBDV_G3 at a multiplicity of infection (MOI) of 0.001. The viral titer reached 10^8^ TCID_50_/mL at 72 h post‑infection (hpi) (Figure 1D), and the replication kinetics of IBDV_G3 in LMH cells are summarized in Table S1.

To characterize the genomic profile of IBDV_G3, the full-length viral genome was amplified and sequenced (Figure 1E). Homology analysis indicated that the VP2, VP3, VP4, and VP5 genes of IBDV_G3 shared the highest nucleotide identities of 94.9%, 94.7%, 93.8%, and 97.3%, respectively, with the serotype 2 A0-lineage reference strain 23/82. In contrast, the VP1 gene displayed the highest identity (97.7%) with the serotype 1 IBDV isolate IBD17JL01. Consistent with the homology results, maximum‑likelihood phylogenetic trees based on individual viral genes revealed that VP2‑VP5 of IBDV_G3 clustered within the serotype 2 A0 lineage, whereas VP1 clustered within the B1a classical‑like lineage (Figure S2). These results demonstrated that IBDV_G3 is a novel IBDV reassortant strain, with segment A derived from classic serotype 2 IBDV and segment B originating from B1a classical-like lineage. To further confirm sequence data, we also performed direct PCR amplification and sequencing of both segments A and B from the original, uncultured quail tissue homogenates (Figure S1). The sequences obtained from the original tissues match those of the cultured clone perfectly, confirming the presence of this genomic constellation in the clinical sample. The VP2 and VP1 proteins from the original and uncultured quail tissues showed 99.93% and 100% identity with that of the isolate IBDV_G3.

**Figure 2:**
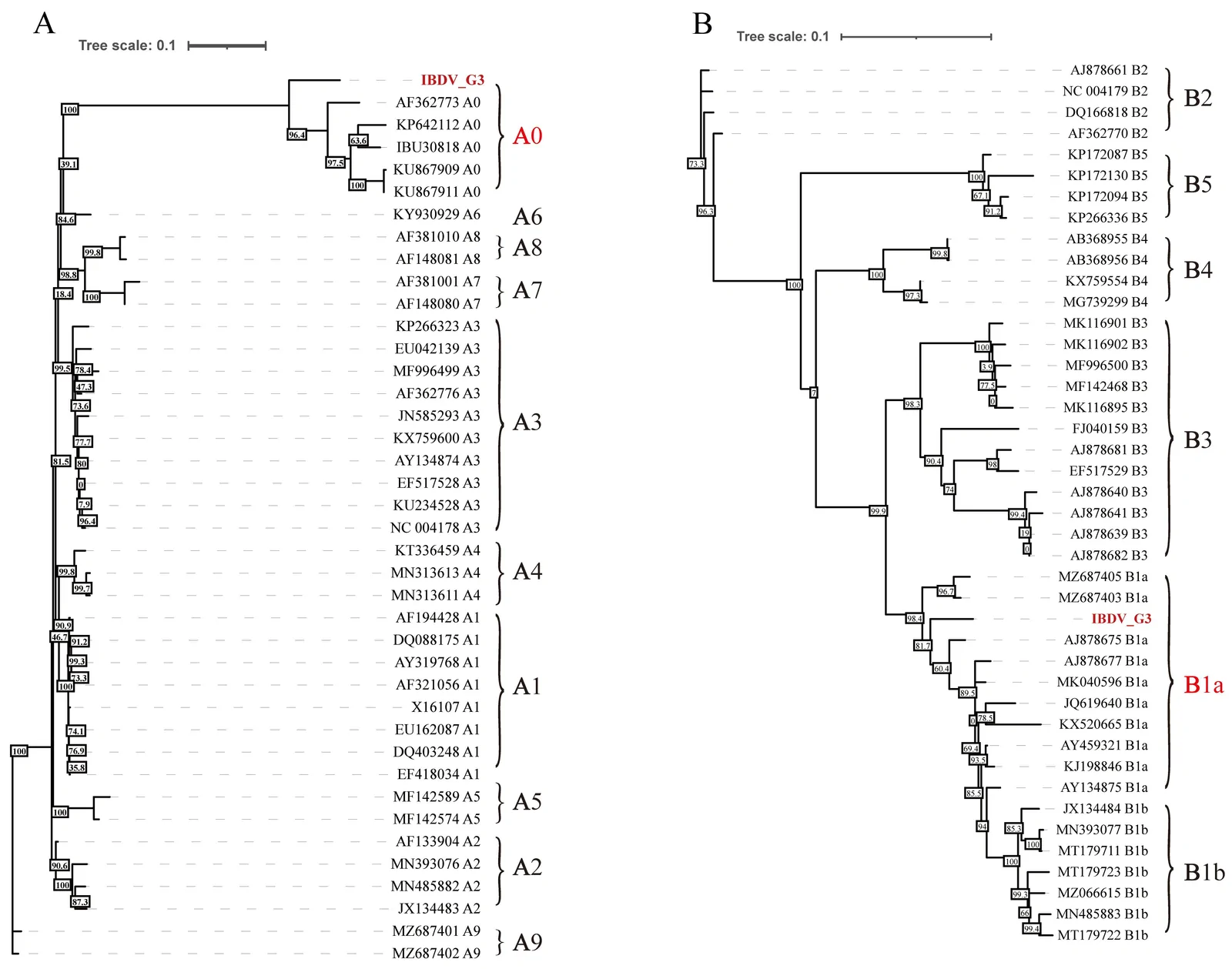
Phylogenetic trees assay for genome of IBDV_G3 Maximum-likelihood (ML) phylogenetic trees inferred from the nucleotide sequences of IBDV_G3 ((**A**) Segment A; (**B**) Segment B). IBDV_G3 strain isolated in this study is highlighted in red, and the reference strains are identified by GenBank accession number.

Phylogenetic analysis further confirmed that segment A of IBDV_G3 clustered within the A0 (serotype 2) sublineage (Figure 2A), whereas segment B grouped with the B1a classical-like sublineage (Figure 2B). The full-length sequences of segment A and segment B of IBDV_G3 have been deposited in NCBI GenBank under the accession numbers PZ896644 (segment A) and PZ896645 (segment B).

## 4. Discussion

In the present study, a novel, naturally reassortant IBDV strain, designated IBDV_G3, was isolated and characterized from diseased quails. Previously reported natural reassortant IBDV strains have been largely confined to A/B segment reassortment within serotype 1, such as the widely circulating A3B1 reassortants (vv-type segment A + classic-type segment B) in Europe and the Middle East [9,10,11], the A3B3 reassortant (vv-type segment A + early Australian-like segment B) reported in Vietnam [12], and the prevalent A2dB1 novel variant in China [13,14]. In contrast, IBDV_G3 carries a serotype-2-related A0 segment A together with a B1a classical-like segment B, representing a B1a classical-like reassortant that has rarely been documented and was recovered from a non-chicken host.

In recent years, global molecular epidemiological surveillance has repeatedly confirmed that reassortment is a key driving force in the current evolution of IBDV: A3B1 reassortants accounted for as high as 81% of strains in British broiler flocks, while the traditional vvIBDV (A3B2) had disappeared [15]. Atypical reassortant genotypes including A3B1, A4B1, and A6B1 were widely prevalent across six countries in the Near East [11]; A3B1 reassortants similarly predominated in Turkish poultry flocks [10]. A 2023–2024 epidemiological survey in China revealed that nVarIBDV accounted for 69.9% of strains and that all vvIBDV isolates were mutated vvIBDV (mvvIBDV) [14]. Collectively, these lines of evidence demonstrate that genomic reassortment and antigenic variation of IBDV are reshaping its global epidemiological landscape. Furthermore, key mutations in the hypervariable region of VP2—including D279Y/N, A222V, and S254G—have been repeatedly identified in reassortant strains from multiple regions. In vitro experiments have confirmed that D279Y is the earliest escape mutation to emerge under vaccine-induced antibody selection pressure [11], while surveillance in Shandong Province has identified the novel A222V substitution as a potential emerging molecular signature of Chinese vvIBDV populations [16], suggesting that vaccine immune pressure may play an important role in the evolution and selection of reassortant strains.

IBDV_G3 was capable of efficient replication in LMH cells without prior cell adaptation, reaching a viral titer of 10^8^ TCID_50_/mL at 72 h post-infection. This property distinguishes it from both classic serotype 1 and serotype 2 IBDV strains, suggesting that this particular reassortment may have conferred unique cellular tropism and replication fitness upon the virus. Notably, IBDV_G3 can be efficiently recognized by mAb 5G10, which targets the conserved B cell epitope ^343^PGALRP^348^ on VP2 of serotype 1 IBDV. This observed cross-reactivity indicates that the targeted epitope is retained in IBDV_G3.

Early studies suggested that quails are refractory to IBDV serotype 1, as inoculated quails developed neither lesions nor antibodies [17]. However, recent research indicates that quails can be subclinically infected and may serve as silent reservoirs. Although direct experimental evidence of productive quail-to-chicken transmission remains limited, serological observations from earlier studies suggest that such cross-species transmission represents a plausible epidemiological risk [8,18]. These observations emphasize that quail–chicken interfaces warrant continuous surveillance for IBDV reassortment and evolutionary variation.

## 5. Conclusions

In conclusion, the present study identifies and characterizes a novel reassortant IBDV strain (IBDV_G3) isolated from quails. IBDV_G3 carries segment A belonging to the serotype-2-related A0 sublineage, while segment B clusters within the B1a classical-like sublineage, and a definitive assignment of segment B to serotype 1 cannot be made based solely on current phylogenetic evidence. Notably, IBDV_G3 can efficiently replicate in LMH cells without adaptive cultivation. Our findings suggest that quails may harbor reassortant IBDVs, highlighting that quail–chicken interfaces warrant enhanced surveillance for IBDV genomic reassortment and evolutionary variation. Further work is needed to explore whether IBDV_G3 could serve as a vaccine candidate, which would require comprehensive assessments.

## Data Availability

The original contributions presented in this study are included in the article/Supplementary Materials. Further inquiries can be directed to the corresponding authors.

## Supplementary Materials

The following supporting information can be downloaded at https://www.mdpi.com/article/10.3390/vetsci13090923/s1, Table S1: Growth curve of IBDV_G3 in LMH cells; Figure S1: PCR amplification of the genome of liver homogenate; Figure S2: Phylogenetic trees assay for genome of IBDV_G3 Maximum-likelihood (ML) phylogenetic trees inferred from the nucleotide sequences of IBDV_G3 ((A) VP1; (B) VP2; (C) VP3; (D) VP4; (E) VP5); Figure S3: Original raw images of PCR gels presented in Figure 1.

## Abbreviations

The following abbreviations are used in this manuscript:

IBD	Infectious bursal disease
IBDV	Infectious bursal disease virus
LMH	Leghorn Male Hepatoma
FBS	Fetal Bovine Serum
DMEM/F12	Dulbecco’s Modified Eagle Medium/Ham’s F-12
CPE	Cytopathic Effect
IFA	Immunofluorescence Assay
FITC	Fluorescein Isothiocyanate
MOI	Multiplicity of Infection
Hpi	Hours Post Infection
TCID_50_	Tissue Culture Infectious Dose 50%
IBV	Infectious Bronchitis Virus
AIV	Avian Influenza Virus
ARV	Avian Reovirus
MG	Mycoplasma Gallisepticum
MS	Mycoplasma Synoviae
NDV	Newcastle disease virus
FAdV	Fowl Adenovirus
TEM	Transmission electron microscope
PBS	Phosphate-buffered saline
