# Supplementary material for: A Naturally Occurring Novel Reassortant Strain of Infectious Bursal Disease Virus from Quails
Source: Vet Sci. 2026 Sep 8;13(9):923. doi: 10.3390/vetsci13090923 (PMC13611912; doi:10.3390/vetsci13090923)

Supplementary Materials

Table S1. Growth curve of IBDV_G3 in LMH cells.

Sample ID	-1	-2	-3	-4	-5	-6	-7	-8	Total	Volume(mL)	Log10 Titer/mL	Anti-Log
G3 24 hpi-1	4	0	0	0	0	0	0	0	4	0.1	2.5	3.16x10^2
G3 24 hpi-2	4	2	0	0	0	0	0	0	4	0.1	3	1x10^3
G3 24 hpi-3	4	1	0	0	0	0	0	0	4	0.1	2.667	4.65x10^2
G3 48 hpi-1	4	4	4	4	4	1	0	0	4	0.1	6.667	4.65x10^6
G3 48 hpi-2	4	4	4	4	1	0	0	0	4	0.1	5.667	4.65x10^5
G3 48 hpi-3	4	4	4	4	2	0	0	0	4	0.1	6	1x10^6
G3 72 hpi-1	4	4	4	4	4	4	4	0	4	0.1	8.5	3.16x10^8
G3 72 hpi-2	4	4	4	4	4	4	4	0	4	0.1	8.5	3.16x10^8
G3 72 hpi-3	4	4	4	4	4	4	0	0	4	0.1	7.5	3.16x10^7
G3 96 hpi-1	4	4	4	4	4	4	4	1	4	0.1	8.667	4.65x10^8
G3 96 hpi-2	4	4	4	4	4	4	2	0	4	0.1	8	1x10^8
G3 96 hpi-3	4	4	4	4	4	4	0	0	4	0.1	7.5	3.16x10^7
G3 120 hpi-1	4	4	4	4	4	4	2	0	4	0.1	8	1x10^8
G3 120 hpi-2	4	4	4	4	4	4	2	2	4	0.1	8.5	3.16x10^8
G3 120 hpi-3	4	4	4	4	4	4	4	1	4	0.1	8.667	4.65x10^8

Figure S1. PCR amplification of the genome of liver homogenate; Line M: 2,000 bp marker, line 1: Segment A of liver homogenate (3262 bp), line 2: Segment B1 of liver homogenate (1364 bp), line 3: Segment B2 of liver homogenate (1500 bp).

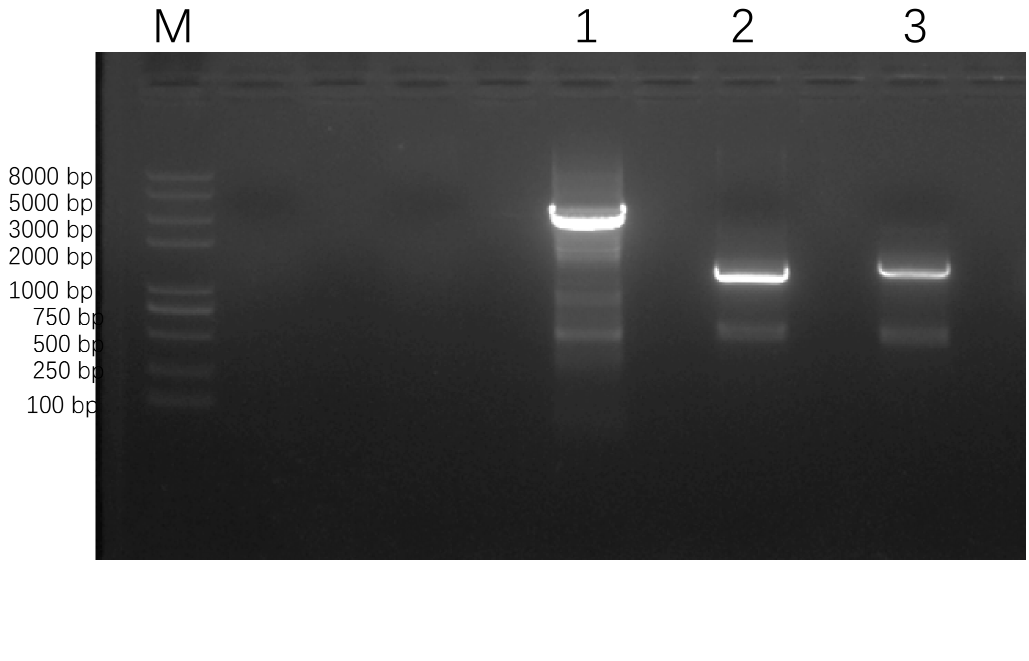

Figure S2. Phylogenetic trees assay for genome of IBDV_G3 Maximum-likelihood (ML) phylogenetic trees inferred from the nucleotide sequences of IBDV_G3 (A: VP1; B: VP2; C: VP3; D: VP4; E: VP5). IBDV_G3 strain isolated in this study is highlighted in red, and the reference strains are identified by GenBank accession number.

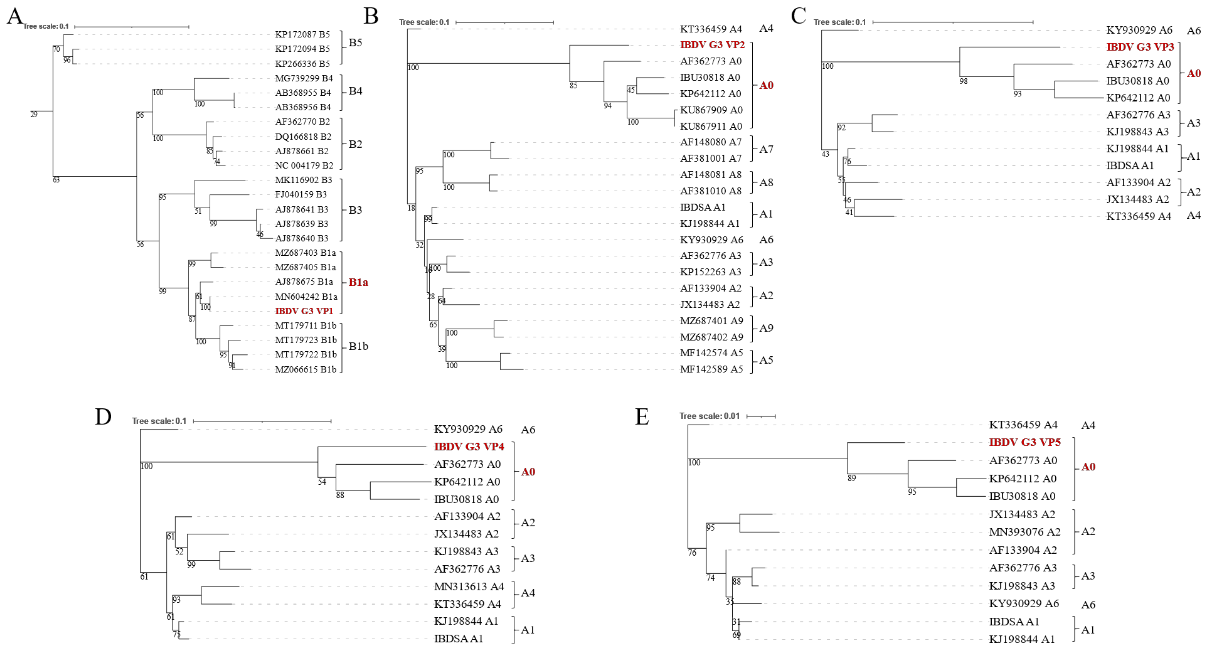

Supplement: Supplementary file 1 [file vetsci-13-00923-s001.zip › Supplementary Materials.pdf]
